# High induction rate onto extended-release naltrexone for people with opioid use disorder: experiences from a Norwegian naturalistic study

**DOI:** 10.1186/s13722-025-00576-9

**Published:** 2025-06-16

**Authors:** Jon Mordal, Farid Juya, Line Holtan, John-Kåre Vederhus, Arild Opheim, Ida H. Brenna, Asle E. Enger, Bente Weimand, Kristin Klemmetsby Solli, Lars Tanum

**Affiliations:** 1 Division of Mental Health and Addiction, Vestfold Hospital Trust, Tønsberg, 3103 Norway; 2 Addiction Unit, Sørlandet Hospital HF, Kristiansand, 4604 Norway; 3 Department of Addiction Medicine, Haukeland University Hospital, Østre Murallmenningen 7, Bergen, 5021 Norway; 4 Department of Clinical Psychology, Faculty of Psychology, University of Bergen, Bergen, 5015 Norway; 5 Department of Addiction Treatment, Oslo University Hospital, Oslo, 0424 Norway; 6 Mental Health Services, Akershus University Hospital, Lørenskog, 1478 Norway; 7 Center for Mental Health and Substance Abuse, University of South- Eastern Norway, Drammen, 3040 Norway; 8 Norwegian Centre for Addiction Research, University of Oslo, Oslo, 0315 Norway

**Keywords:** Opioid use disorder, Extended-release naltrexone, Medically managed withdrawal, Treatment induction

## Abstract

**Background:**

For people with opioid use disorder (OUD), extended-release naltrexone (XR-NTX) is an effective antagonist treatment option. However, successful opioid tapering and abstinence is a prerequisite for XR-NTX induction and has repeatedly been reported as a major barrier to effective treatment. The aims of this study were to describe XR-NTX induction rates, reasons for incomplete induction, and extraordinary complications reported during the induction phase. We also compared sociodemographic and clinical variables among those who did and did not complete induction onto XR-NTX.

**Methods:**

This naturalistic, multicenter, and open-label Norwegian study of XR-NTX included men and women aged 18–65 who had severe OUD. Most participants were referred to inpatient medically managed opioid withdrawal and received individualized pharmacological and psychosocial treatment according to clinical assessment and national guidelines. After opioid withdrawal, the participants underwent a minimum of three opioid-free days prior to XR-NTX induction. Variables were collected through baseline assessments and a retrospective patient chart review. XR-NTX induction completers and non-completers were compared via bivariate and logistic regression analyses.

**Results:**

Of 129 participants with recent opioid use at inclusion, 106 (82%) completed XR-NTX induction. Induction was initiated in an inpatient setting for 116 participants (90%) and extraordinary complications were noted for 19 (15%) patients. Withdrawal symptoms and ambivalence were the most common reasons for non-completion, each noted in 75% of the cases. As compared with those who successfully completed induction, non-completers more often reported lifetime hepatitis (78% vs. 52%, *p* = 0.017), had a longer period of current substance use (mean 119 vs. 54 months, *p* = 0.001), and more frequently used methadone prior to study inclusion (43% vs. 8%, *p* < 001). In logistic regression analyses, methadone use was the only significant factor and was negatively associated with completion (odds ratio 0.20, 95% confidence interval = 0.05–0.72, *p* = 0.014).

**Conclusion:**

The results demonstrate the safety, efficacy and tolerability of a Norwegian opioid withdrawal and XR-NTX induction procedure. Although the present induction rate was high, our findings indicate that methadone users need special attention and tailored interventions regarding opioid withdrawal management and XR-NTX induction.

**Trial registration:**

The study is registered at clinicaltrials.gov (NCT03647774).

## Background

Opioid use disorder (OUD) has a number of negative consequences for patients, their relatives, and to society, and overdose from heroin and other opioids is a leading cause of death among drug users [1–3]. Opioid agonist treatment (OAT) with methadone or buprenorphine is available in many countries, but additional treatment options are needed to increase the number of people with OUD in treatment [4]. Previously, naltrexone (an opioid antagonist) was available only in tablet form, with dubious effectiveness in patients with OUD [5]. The introduction of extended-release naltrexone (XR-NTX) as an intramuscular injection agent has provided a viable alternative to OAT: XR-NTX offers a four-week-long opioid-blocking effect, which presents a realistic choice for individuals with OUD who are seeking treatment [6–8], up to one-third of whom may prefer naltrexone [9]. XR-NTX is, however, approved for clinical use in only a few countries, such as the U.S. and Russia, and successful opioid tapering as well as abstinence is a prerequisite for XR-NTX induction, which has repeatedly been reported as a major barrier to effective treatment [6].

Symptoms of opioid withdrawal syndrome include, for example, irritability, pains, and diarrhea; their onset and duration vary with the half-life of the opioid used and the duration of regular use. Medically managed opioid withdrawal is provided in different settings, but procedures are generally not adapted to the needs of patients inducted onto XR-NTX [10]. For opioid-using patients, naltrexone can precipitate severe and acute withdrawal, and the prescribing information therefore recommends that patients are opioid-free for at least seven to 10 days before receiving their first injection [11]. This delay minimizes the risk of naltrexone-induced withdrawal and may optimize both treatment induction and retention, although it may be challenging for patients and carries a significant risk of treatment discontinuation as well as discharge against medical advice.

According to a systematic review of the XR-NTX literature, different initiation procedures have been proposed, and successful XR-NTX induction rates vary from 7% to 70% among outpatients and 56% to 89% among inpatients [6]. Gradual agonist tapering has been commonly applied, with a minimum of three or four opioid-free days prior to induction. Rapid induction procedures (between five and six days) suggest less use of agonist tapering and more use of non-opioid medication, including gradual titration of small doses of oral naltrexone [12, 13].

Few studies have explored the associations between patient characteristics and induction rates. In a U.S. study of a rapid inpatient procedure, completed induction was associated with older age, less opioid use per day, and absence of comorbid non-opioid substance use at baseline [14]. A recent paper summarized five U.S. studies on rapid XR-NTX induction from both in- and outpatient settings [15]. The authors found a higher induction rate among inpatients (65%) versus outpatients (48%) and concluded that inpatient settings may be better for opioid users with greater severity, including heroin or intravenous use, and outpatient induction is more likely to succeed for prescription opioid users.

Additional knowledge is needed regarding XR-NTX initiation performed with different procedures in various settings and regions as well as patient characteristics that predict successful induction [4, 6]. Thus, this paper has the following research aims:

1. To describe XR-NTX induction rates from a naturalistic, multicenter study in Norway (NaltRec).
2. To detail the study setting, extraordinary complications reported during the induction phase, and registered reasons for incomplete induction.
3. To compare sociodemographic and clinical variables among participants who did and did not complete induction onto XR-NTX.

## Methods

The NaltRec study was a 24-week naturalistic, multicenter, open-label trial of XR-NTX that offered an optional 28-week treatment extension [16]. Participants were recruited from in- and outpatient units at five urban addiction clinics in Norway; patients received 380 mg XR-NTX intramuscularly (Vivitrol) every fourth week during the study period. XR-NTX in Norway was available only in clinical trials, and inclusion dates were from September 2018 to September 2020. This paper focuses on treatment induction.

### Inclusion and exclusion criteria

Inclusion criteria for the NaltRec study were men and women aged 18–65 with severe OUD, as defined by the Diagnostic and Statistical Manual of Mental Disorders, 5th edition criteria. The sample presented in this paper consists of participants with recent opioid use (i.e. use within one week prior to study inclusion). Some participants were in OAT before study inclusion; those who were not in OAT were enrolled in the program at inclusion to ensure immediate access to agonist treatment and relevant health services after study discontinuation, if needed. Exclusion criteria were pregnancy, lactation, acute alcoholism, and severe medical or psychiatric disease that could interfere with study participation, such as decompensated hepatic cirrhosis, renal failure, HIV with related symptoms, current or recurrent affective disorders with suicidal behavior, or psychotic disorders. A physician examined participants for serious medical diseases.

### Routine opioid withdrawal management

In line with the study protocol, most eligible participants were referred to routine inpatient medically managed opioid withdrawal and were included on the day of admission. However, and after individual assessment, recruitment and study entry were also possible from other settings, such as outpatient and long-term treatment units. Admitted participants received individualized pharmacological and psychosocial treatment according to clinical assessment and national guidelines [17], commonly with standard buprenorphine taper: After development of mild-to-moderate withdrawal symptoms, sublingual buprenorphine was gradually administered and then tapered over 3 to 10 days, often with adjunctive medications such as clonidine [17].

### XR-NTX treatment induction

After opioid cessation and according to the study protocol, the participants underwent a minimum of three days without opioid intake, which was followed by a urinary drug screening and a challenge test of 0.4 mg naloxone intramuscular injection. Participants who had an opioid-negative urine test and passed the naloxone challenge test without experiencing increased withdrawal symptoms received an intramuscular injection of 380 mg XR-NTX in the gluteal muscle. Participants were typically discharged from the inpatient units within one to three days after the initial XR-NTX dose. After induction, all patients were monitored by study personnel every fourth week through clinical interviews, urine drug tests, and new injections. For participants who did not complete induction, follow-up with OAT was recommended.

### Assessments

Upon signing the informed consent, patients provided their baseline demographics and information regarding substance use, physical and mental health, and education using the Addiction Severity Index [18]. Additional baseline assessments of mental health were performed with the Hopkins’ Symptom Checklist 25 (SCL-25) [19] and the Adult ADHD Self-Report Scale 18-item version (ASRS-18) v1.1 [20]. The 18 questions were dichotomized according to Kessler et al.’s description, and a positive score of nine or higher was considered to be a clinically significant symptom level. Additionally, the first author retrospectively collected variables by reviewing hospital charts from the study entry process. Variables included any recent substance use within one week prior to study inclusion (based on self-report and/or by urine drug tests), registered reasons for incomplete induction, and clinical complications for all participants during the induction phase (two weeks before and after the first injection). The first author evaluated all complications to identify extraordinary cases, which typically included severe withdrawal symptoms and acute medical or psychiatric conditions. For participants who completed induction, registered serious adverse events during the first 14 days were collected from the NaltRec electronic case report form and were also defined as extraordinary complications.

### Statistical analyses

Descriptive statistics were used to describe the study sample. Symmetrically distributed continuous variables were reported as mean ± standard deviation (SD). Continuous variables that were not symmetrically distributed were reported by median and range values. Study participants who either completed (completers) or did not complete (non-completers) XR-NTX induction were compared using Pearson’s chi-squared or Fischer Exact tests for categorical variables and Student’s t-test or the Mann–Whitney U test for continuous variables. Significance level was set to *p* < 0.05. We then performed a logistic regression analysis in which complete versus incomplete induction was the outcome variable. The significant variables identified in the bivariate analyses were included as independent variables, and odds ratios (OR) were reported with 95% confidence intervals (CI). All analyses were performed using SPSS, version 27.

## Results

**Fig. 1:**
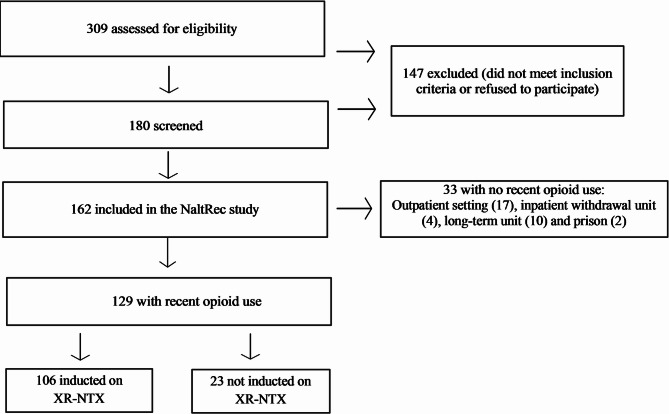
Flowchart for inclusion of study participants NaltRec study: “*Enablers and hindrances for longer-term abstinence in opioid dependent individuals receiving treatment with extended-release naltrexone: A Norwegian longitudinal recovery trial*” Recent opioid use: Any use < one week prior to study inclusion, based on self-report and/or by urine drug tests

Among 309 participants assessed for eligibility, 180 were screened and 162 were included in the NaltRec study (Fig. 1). Baseline participant characteristics and 6-month treatment outcomes of the NaltRec study have been previously reported [21, 22]. The study sample presented in this paper comprised 129 participants who had recently used opioids and required withdrawal management at the time of inclusion. Among them, 106 participants (82%) successfully completed XR-NTX induction. The number of included participants with recent opioid use at each of the five study sites varied from 10 to 49, with induction rates ranging from 70 to 91% (*p* = 0.311). Most participants (*n* = 105) were included before the COVID-19 pandemic in March 2020, and induction rates did not change significantly before and during the pandemic (84% vs. 75%, *p* = 0.309). The participants had a mean age of 38.3 years (SD 10.2, range 21–63), 34 (26%) were women, and 90 (70%) were in OAT prior to study inclusion. The average duration of education was 11.9 years (SD = 2.6, range 5–19).

XR-NTX induction was initiated in an inpatient setting for 116 participants (90% of 129), (Table 1). Of the 87 participants who were inducted during inpatient opioid withdrawal management, 76 had a planned study-related admission (two were admitted twice); 11 were informed about the study, screened, and included during routine hospitalizations. Four outpatients were inducted during the COVID pandemic, and in three of these cases, infection prevention was one of the reasons for outpatient induction. The mean number of opioid-free days before the first injection was 6.6 (SD 3.6, range 1–22).

**Table 1:** Study setting and extraordinary complications during withdrawal management and XR-NTX induction

	Recent opioid use (*n* = 129)	Extraordinary complications (*n* = 19)
**Inducted on XR-NTX**	**106 (82%)**	
Outpatient setting	13 (10%)	
After a recent hospitalization for withdrawal management*	9	*2*
Without a recent hospitalization	4	*0*
Inpatient setting	93 (72%)	
Inpatient withdrawal management	87	*14*
Long-term treatment unit	6	*3*
**Not inducted on XR-NTX**	**23 (18%)**	
Inpatient withdrawal management	23	*0*

Recent opioid use: Within one week prior to study inclusion, based on self-report and/or by urine drug tests. Extraordinary complications include serious adverse events

*Hospitalization for inpatient withdrawal management within eight weeks prior to induction

Extraordinary complications and / or serious adverse events during induction and the first 14 days were noted for 19 participants (15% of 129); these included both inpatient (*n* = 17) and outpatient (*n* = 2) inductions, both before and after the first XR-NTX injection (Tables 1 and 2). All complications were nonlethal and included severe withdrawal symptoms (*n* = 7), medical (*n* = 5) and psychiatric (*n* = 3) complications, and two others (a severe overdose and use of illegal drugs during hospitalization). Two participants with medical complications (seizures and gastroenteritis with fever) were subsequently transferred to medical units. The prevalence of extraordinary complications at each of the five study sites varied from 4 to 24% (*p* = 0.254). Extraordinary complications were not observed among non-completers, and only among one of the 19 participants with recent use of methadone at admission.

**Table 2:** Extraordinary complications during withdrawal management and XR-NTX induction (*n* = 19)

Details of complication	*n*
**Inpatient induction (** ***n*** ** = 17)**	
Before injection only	
Severe withdrawal symptoms as well as verbal or physical agitation (W)	2
Other difficult medical conditions (diabetes and hypertension) (S)	2
Severe nonlethal overdose and burn injury while on home leave during hospitalization (O)	1
Use of illegal drugs during hospitalization (O)	2
Before and after injection	
Severe withdrawal symptoms and verbal agitation (W)	1
Paranoid psychotic symptoms before and after injection (P)	1
After injection only	
Severe withdrawal (W)	1
Severe withdrawal symptoms and immediate relapse after injection (W)	1
Relapse and readmission for inpatient withdrawal management (W)	2
Medical complications with diabetes (M)	1
Seizures after injection that were treated in the present unit (M)	1
Seizures after injection and transfer to a medical unit (M)	1
Depression and increased suicidal ideation (P)	1
**Outpatient induction (** ***n*** ** = 2)**	
After injection	
Gastroenteritis and fever, followed by admission to a medical unit (M)	1
Psychotic symptoms (P)	1

Extraordinary complications include serious adverse events (*n* = 6) and are categorized as withdrawal symptoms (W), medical (M) or psychiatric (P) complications, and others (O)

All the 23 non-completers were admitted to inpatient withdrawal units (two were admitted twice). Withdrawal symptoms and ambivalence were the most common reasons for non-completion, each noted in 75% of the cases (Table 3). For the 110 participants admitted to medically managed opioid withdrawal, the mean length of stay was 10.5 days for non-completers (SD 8.1, median 7.0, range 1–34) and 11.9 days for completers (SD 6.0, median 11.0, range 2–28, *p* = 0.222). Eleven non-completers were discharged with opioid agonist medication and transferred to an OAT program, whereas 10 were enrolled in OAT prior to inclusion and were recommended to continue the treatment. Two non-completers were discharged against medical advice with no established follow-up appointments.

**Table 3:** Reasons for incomplete XR-NTX induction as registered in hospital records (*n* = 23)

Reason	*n*
Withdrawal symptoms	5
Withdrawal symptoms and ambivalence	10
Withdrawal symptoms, ambivalence, and pain	3
Ambivalence and side effects of naltrexone tablets	1
Ambivalence, change of mind, or not otherwise specified	3
Discharged due to inpatient illegal substance use	1

Comparing completers (*n* = 106) and non-completers (*n* = 23), no significant differences were seen regarding setting, age, gender, or years of education (Table 4). Non-completers more often reported lifetime hepatitis than completers (78% vs. 52%, *p* = 0.017), whereas no significant differences were observed in mental health variables (i.e., according to the ASRS and H-SCL-25). The groups did not differ in their perceived most problematic substance, but non-completers had a longer period of current substance use (mean 119 vs. 54 months, *p* = 0.001) and were more likely to have recently used methadone prior to study inclusion, regardless of whether it was prescribed (43% vs. 8%, *p* < 001).

**Table 4:** Comparison via bivariate analyses of completers and non-completers (*n* = 129)

Background information	Inducted on XR-NTX (*n* = 106)	Not inducted on XR-NTX (*n* = 23)	*P*
**Setting**			
Study site (% of participants at five sites, range)	70% − 91%	9% − 30%	*0.311*
Outpatient inclusion, *n* (%)	13 (12)	0 (0)	*0.123*
**Sociodemographic variables**			
Age, mean (SD)	38.0 (± 9.8)	39.4 (± 12.1)	*0.707*
Female gender, *n* (%)	27 (26)	7 (30)	*0.624*
Years of education, mean (SD)	12.0 (± 2.4)	11.2 (± 3.2)	*0.076*
**Physical and mental health (baseline)**			
Body mass index, mean (SD)	24.0 (± 4.1)	26.1 (± 5.3)	*0.121*
Chronic medical illness, *n* (%)	40 (38)	8 (35)	*0.904*
Positive hepatitis, lifetime, *n* (%)	55 (52)	18 (78)	*0.017*
H-SCL-25 score, mean (SD)	2.0 (± 0.6)	2.0 (± 0.6)	*0.777*
ASRS above clinical cutoff, *n* (%)	40 (38)	10 (43)	*0.542*
**Substance use**			
Opioids, age at onset, mean (SD)	20.4 (± 5.6)	18.6 (± 5.9)	*0.107*
OAT prior to study inclusion, *n* (%)	71 (67)	19 (83)	*0.210*
Most problematic substance (baseline, self-report)			
Heroin, *n* (%)	49 (46)	7 (30)	*0.346*
Methadone or buprenorphine, *n* (%)	20 (19)	8 (35)	
Multiple substances, *n* (%)	26 (25)	5 (22)	
Others, *n* (%)	11 (10)	3 (13)	
Months of current use, mean (SD)*	53.5 (± 79.5)	119.1 (± 122.6)	*0.001*
Substance use within one week prior to study inclusion**			
Heroin, *n* (%)	49 (46)	11 (48)	*0.982*
Buprenorphine, *n* (%)	70 (66)	11 (48)	*0.101*
Methadone, *n* (%)	9 (8)	10 (43)	*< 0.001*
Cannabis, *n* (%)	45 (42)	13 (57)	*0.165*
(Meth)amphetamines, *n* (%)	30 (28)	8 (35)	*0.485*
Benzodiazepines, *n* (%)	68 (64)	16 (70)	*0.336*
Alcohol, *n* (%)	15 (14)	2 (9)	*0.735*
Number of substances used, mean (SD) (range 1–6)***	2.7 (± 1.3)	3.2 (± 1.5)	*0.235*
Injection drug use, *n* (%)	39 (37)	12 (52)	*0.139*

ASRS-18 Adult ADHD Self-Report Scale 18-item version, SCL-25 Hopkins’ Symptom Checklist 25, OAT Opioid agonist treatment. Groups were compared using Pearson’s chi-squared or Fischer Exact tests for categorical variables and Student’s t-test or the Mann–Whitney U test for continuous variables

*n = 125 due to missing data for four participants

**Based on patients’ self-report or results of urine toxicology (regardless of prescription)

***The seven aforementioned substances are summarized

A logistic regression model included lifetime hepatitis, months of current substance use, and recent use of methadone. The full model was statistically significant (chi-squared (*n* = 125) = 15.90, *p* = 0.001), indicating that the model was able to distinguish between completers and non-completers, correctly classifying 84% of the cases. As presented in Table 5, only recent use of methadone made a unique, significant contribution to the model, which demonstrated that those who recently used methadone were less likely to complete XR-NTX induction (OR 0.20, 95% CI = 0.05–0.72, *p* = 0.014). The analysis was repeated with age and sex as additional variables, and the results remained nearly identical.

**Table 5:** Multivariate logistic regression predicting likelihood of completing XR-NTX induction (*n* = 125)

Variable	OR*	95% CI*	*P*
Positive hepatitis, lifetime	0.36	(0.10–1.25)	*0.107*
Months of current substance use	0.99	(0.99–1.00)	*0.404*
Methadone use within one week prior to study inclusion	0.20	(0.05–0.72)	*0.014*

OR Odds ratio, CI Confidence interval, *n* = 125 due to missing data for four participants

## Discussion

The main findings in this naturalistic, multicenter study were that the XR-NTX induction rate was high (82%), and methadone use was negatively associated with completion.

The induction rate was higher than in many other studies [6] but was comparable with recent randomized trials from Norway [8] and the US [23]. Multiple reasons may explain this high induction rate. Most participants were probably significantly motivated because XR-NTX was not clinically available in Norway at the time, whereas in this study, it was offered as a medication at no cost. Although OAT is also offered at no cost in Norway, the participants chose to test the alternative antagonist medication. Additionally, the presence of dedicated and experienced study and health staff who had previously conducted a Norwegian randomized trial of XR-NTX [8] may have been a significant factor. Their experience provided them with a thorough understanding of the recruitment process, enabling them to confidently recommend the medication based on the positive outcomes observed in the prior study and to facilitate treatment induction. The study applied an individualized and flexible induction process based on user involvement, which enabled a high inclusion rate even during the COVID-19 pandemic. Most participants were referred to inpatient medically managed opioid withdrawal (some were admitted twice), which allowed extensive, individualized pharmacological and psychosocial treatment that was fully funded by the public health system. The average inpatient length of stay was approximately 12 days, which is comparable with procedures in a large U.S. study [24].

Our finding that recent methadone use was negatively associated with completion has, to our knowledge, not been explicitly reported in previous XR-NTX papers. However, a U.S. pilot trial (*n* = 47) found that baseline methadone use was associated with shorter retention in oral naltrexone treatment [25]. Additionally, a large U.S. study of XR-NTX (*n* = 283) found that the use of agonist medications (methadone or buprenorphine) during opioid withdrawal treatment was linked to induction non-completion [24]. Most U.S. studies on XR-NTX have focused on illegal opioid users, and participants with regular baseline use of methadone or buprenorphine have typically been excluded [12, 15, 23]. However, individuals in OAT may be interested in receiving XR-NTX [9, 26] and were included in the present NaltRec study, comprising two-thirds of all participants. The study overall reported high treatment satisfaction, low cravings for heroin, and a significant decrease in opioid use at the 24-week follow-up [21].

Regarding induction challenges, methadone is a full opioid agonist and has been connected to more intense and prolonged withdrawal symptoms than the partial agonist buprenorphine in both quantitative [27] and qualitative [28] studies. This also aligns with clinical experience from OAT and withdrawal management settings; it has often been observed that methadone users find it substantially difficult to complete opioid tapering and abstinence, probably due to its agonist and broad clinical effects. Many patients report that methadone has positive effects on several symptoms, such as substance craving, anxiety, and physical pain, as described in a qualitative study [28], which may increase the challenge of tapering and abstaining. The prevalence of methadone use among OAT patients varies across nations and regions, with 61% in a recent US study [29] and 30% in a Norwegian national cohort [30]. Many of these patients may prefer XR-NTX if it were available. Our findings indicate that methadone users need special attention and tailored interventions regarding XR-NTX induction, and these may include extra psychosocial support [31, 32], extended methadone tapering, more intensive symptomatic treatment, delayed naltrexone induction, and inpatient services. Additionally, protocols with gradual titration of small doses of oral naltrexone demonstrate promising alternatives for increased XR-NTX induction rates [12, 13], which may also apply to methadone users. The standard buprenorphine tapering protocol commonly used in this study necessitates the presence of opioid withdrawal symptoms before initiation, which can be intolerable for many patients. Gradual dosing strategies for buprenorphine induction also show promise, but there is a need for prospective, randomized, controlled trials [33].

Despite extraordinary complications being noted for 14% of the participants, the results demonstrate the safety and tolerability of a Norwegian opioid withdrawal and XR-NTX induction procedure. Methodological differences challenge comparisons across studies. “Adverse events” were frequently reported in an outpatient U.S. study of XR-NTX induction (55.6% for rapid and 76.5% for standard procedures) [13], whereas a recent inpatient U.S. study reported infrequent “safety events” (5.3% for rapid and 2.1% for standard procedures) [12]. However, both studies concluded that the procedures were safe, efficient and tolerable. Nevertheless, opioid withdrawal and XR-NTX induction may be challenging for patients, their relatives, and health personnel, which is illustrated by qualitative data derived from this study [34], in which one participant vividly described his induction experience: “It felt like being on a speeding train that suddenly crashed into a solid rock wall at a velocity of 360 km/h.” Among extraordinary complications noted in the present study were severe psychosis, a severe nonlethal overdose, and seizures; and two participants were immediately transferred to medical units, one of whom was inducted as an outpatient. Outpatient approaches are cost effective and feasible for XR-NTX induction [6], but there are considerable geographical variations in the organization and quality of such services. Our study highlights the importance of careful planning and a personalized approach when conducting outpatient induction; easy access to round-the-clock emergency services that can provide immediate support are preferable. Although inpatient settings have been recommended for rapid induction procedures [15], such treatment is expensive and may not be available or feasible, and many participants may also prefer outpatient services, if possible.

The causes of the observed complications were probably numerous and multifaceted. Generally, complications observed prior to XR-NTX induction were likely related to opioid withdrawal, while those occurring after the injection were likely due to precipitated withdrawal and/or the side effects of naltrexone. Surprisingly, there was no association between extraordinary complications and recent methadone use. Although patients with severe medical or psychiatric diseases were excluded from participation, comorbid substance use and/or mild to moderate medical or psychiatric conditions may have complicated the management of opioid withdrawal. Moreover, the number of opioid-free days before the first injection may be significant for both complications and induction rates. However, this factor has been scarcely investigated.

Reasons for incomplete induction have seldom been described in previous studies, but we found that withdrawal symptoms and ambivalence were most frequently reported. This aligns with a qualitative XR-NTX study in which participants reported detoxification, ambivalence, and fears regarding antagonist treatment to be barriers to treatment initiation [35]. Our study was not designed to assess potential interventions for withdrawal symptoms and ambivalence. However, many of the non-completers had recently used methadone and thus probably experienced challenging withdrawal symptoms, as discussed above. Non-completers also had a long current period of substance use at inclusion (a mean of 119 months), which may explain some of their challenges regarding withdrawal and ambivalence. Additionally, XR-NTX treatment was not approved for clinical use in Norway and thus was unknown to most participants. In case of a future clinical approval, XR-NTX will be better known, and thus the level of ambivalence among eligible patients may be reduced. Nevertheless, addressing and resolving ambivalence has long been a key feature in substance use treatment, such as in motivational interviewing [32]. Resolving ambivalence may be even more important in antagonist treatment, as this often implies major behavioral and emotional changes [7]. Notably, two of the non-completers were discharged against medical advice with no established follow-up appointments, which placed them at an increased risk of overdose and demonstrates the need for feasible alternative plans for these patients.

This study has several limitations. A selection bias may have occurred because the study was non-randomized, and highly motivated patients may have been included. Baseline assessments were based on self-reported data, and some variables were collected retrospectively via medical records reviews; as such, they cannot be independently verified. The definition of “extraordinary complications” was not standardized. Structured assessments for withdrawal or mental health symptoms during the induction phase were not performed. Finally, the sample size was relatively small, which limited the number of potential predictors that could have been included in the analyses. However, we employed an exploratory approach and examined several of the most relevant factors that have been identified in previous research.

## Conclusions

The results demonstrate the safety, efficacy, and tolerability of a Norwegian opioid withdrawal and XR-NTX induction procedure, despite the potential complications that can occur in medically managed opioid withdrawal. The induction rate for XR-NTX was high in this naturalistic setting. However, methadone use was negatively associated with completion, underscoring the importance of providing special attention to patients who use methadone. This study contributes to the limited literature on XR-NTX induction, although further research is needed to explore both predictors and different methods for initiating individuals with OUD onto XR-NTX in inpatient and outpatient settings.

## Data Availability

The datasets generated and analyzed during the current study are not publicly available due to the possibility of compromising individual privacy and anonymity but are available from the corresponding author on reasonable request.

## Abbreviations

ASI: Addiction Severity Index
ASRS-18: Adult ADHD Self-Report Scale 18-item version
CI: Confidence interval
OAT: Opioid agonist treatment
OUD: Opioid use disorder
OR: Odds ratio
SCL-25: Hopkins’ Symptom Checklist 25
SD: Standard deviation
XR-NTX: Extended-release naltrexone
